# Fate of Carbohydrates and Lignin during Composting and Mycelium Growth of *Agaricus bisporus* on Wheat Straw Based Compost

**DOI:** 10.1371/journal.pone.0138909

**Published:** 2015-10-05

**Authors:** Edita Jurak, Arjen M. Punt, Wim Arts, Mirjam A. Kabel, Harry Gruppen

**Affiliations:** 1 Wageningen University, Laboratory of Food Chemistry, Bornse Weilanden 9, 6708 WG, Wageningen, The Netherlands; 2 C4C Grondstoffen B.V. Driekronenstraat 6, 6596 MA, Milsbeek, The Netherlands; University Of Helsinki, FINLAND

## Abstract

In wheat straw based composting, enabling growth of *Agaricus bisporus* mushrooms, it is unknown to which extent the carbohydrate-lignin matrix changes and how much is metabolized. In this paper we report yields and remaining structures of the major components. During the Phase II of composting 50% of both xylan and cellulose were metabolized by microbial activity, while lignin structures were unaltered. During *A*. *bisporus’* mycelium growth (Phase III) carbohydrates were only slightly consumed and xylan was found to be partially degraded. At the same time, lignin was metabolized for 45% based on pyrolysis GC/MS. Remaining lignin was found to be modified by an increase in the ratio of syringyl (S) to guaiacyl (G) units from 0.5 to 0.7 during mycelium growth, while fewer decorations on the phenolic skeleton of both S and G units remained.

## Highlights:

- 50% of xylan and cellulose are metabolized in composting.
- During *A*. *bisporus’* mycelium growth 45% of lignin was metabolized.
- S:G ratio of remaining lignin increases from 0.5 to 0.7 during mycelium growth.
- Part of the guaiacyl units of lignin become water soluble during mycelium growth.

## Introduction

In the conventional European process, compost for mushroom growth is produced from a basic mixture (BM) of straw bedded horse manure, wheat straw, poultry manure and gypsum [1]. The BM composition and duration of composting phases can differ in different parts of the world, but compost always serves as carbon and nitrogen source for *Agaricus bisporus’* mushroom growth.

The main ingredient in the European compost, wheat straw, contains about 57% (w/w) of carbohydrates, mostly cellulose (44 mol%) and xylan (46 mol%), and 27% (w/w) of lignin [2]. Cellulose is a non-branched polymer of β-1,4-linked glucosyl units. Xylan in grasses, like wheat straw, is composed of a 1,4-linked β-D-xylopyranosyl-backbone with arabinosyl, O-acetyl and (4-O-methyl-) glucuronic acid side chains [3]. However, the exact amounts and distribution of all substituents on wheat straw xylan is not reported. Lignin is composed of three main monolignols: p-hydroxyphenyl (H), guaiacyl (G) and syringyl (S) phenylpropanoid units. In wheat straw lignin all three units are present (H:G:S ratio of 6:64:30; [4]). Cellulose, xylan and lignin form together a complex, hard to degrade, network. In grasses, like wheat straw, xylan can adsorb to cellulose, but also be oxidatively cross-linked with other xylan molecules and with lignin via hydroxycinnamic acid residues [5]. Composting aims at opening up such a complex to facilitate release of monosaccharides, which serve as carbon source during *A*. *bisporus’* mushroom growth [2]. As such, composting has a similar goal as many other pre-treatments of lignocellulosic plant biomass aiming at an improved enzymatic release of fermentable monosaccharides to produce biofuels and chemicals from. Therefore, insights in the reactions occurring could be of use for other pre-treated plant materials.

The industrial production of compost is carried out in closed tunnels and involves three phases, described in detail elsewhere [2]. In brief, meso- and thermophilic microbiota decompose BM (Phase I (PI)), causing a rise in temperature to 80°C and release of ammonia. In the next phase (PII), microorganisms, in particular actinomycetes and fungi, consume at a maximum of 60°C about 40% of the ammonia present, while the other part disappears in the air [6]. As a result of two composting phases, compost has become accessible and specific for *A*. *bisporus* mycelium growth in the third phase at temperatures around 24°C for 16 days (PIII–16). In PIII, the *A*. *bisporus* mycelium is known to consume (part of) the microbiota present [7]. For optimal growth the *A*. *bisporus* mycelium needs also to degrade and consume the carbohydrates and, possibly, lignin present [1, 2]. PIII–16 compost is considered mature and by adding a casing layer on top of this compost the fruiting body formation starts [6].

Composting is an accelerated version of natural decomposition of lignocellulose by the microorganisms present [8]. The activity of these microorganisms and growth of *A*. *bisporus* chemically alters the compost [1, 2]. Quantification of remaining components, like xylan, cellulose, lignin and protein, however, has not been reported. So far, mainly qualitative changes in compost composition have been reported, with a focus on a decrease in carbohydrate and protein as based on total dry matter [1, 2, 9]. Lignin degradation has been mentioned, but only indirect evidence was shown, either by investigating whether *A*. *bisporus* can grow on radioactive labelled ^14^C lignin or by determining the presence of laccase-activity and manganese peroxidase. The latter is hypothesized to be linked to lignin degradation during mycelium growth [10, 11]. Although important, these results lack the possibility to determine absolute quantities of carbohydrates, lignin and protein metabolized.

In our research, in a tunnel-experiment at industrial scale, a mass balance was conducted for dry matter as well as for proteins, cellulose, xylan, lignin and ash. In addition, the structural changes of xylan and lignin were studied. Mapping the amounts and structures of the main components available for mushroom growth is essential for improving the process. Generally, our study contributes to the understanding how wheat straw compost is degraded.

## Materials and Methods

### Composting process

At the composting company CNC-C4C (Milsbeek, The Netherlands) basic mixture (BM) was obtained by mixing on a wet basis, 63% w/w of fresh horse manure, 2% w/w of gypsum, 1% w/w of ammonium sulphate solution (20% w/v (NH_4_)_2_SO_4_ in water), 17% w/w of filtered percolate water, 11% w/w of chicken manure, 4% w/w straw and 2% w/w of water. Fresh horse manure and wheat straw were collected in October 2013 and the experiment was carried out in October and November 2013. For this experiment, one tunnel was assigned for compost production from which all samples were taken from. The composting phases are described elsewhere [2]. In addition to the previously described information, it should be mentioned that the PI phase lasted for 5 days, and reached 80°C under formation of ammonia, after which PI compost was obtained. To PI compost 10 g kg^−1^ of PII compost was added to introduce viable microflora necessary for the conditioning phase. The duration of PII was also 5 days. To PII compost 4.5 g kg^−1^ of rye-based spawn was added and inoculated for 16 days after which PIII–16 mycelium grown compost was obtained.

### Samples

**Fig 1:**
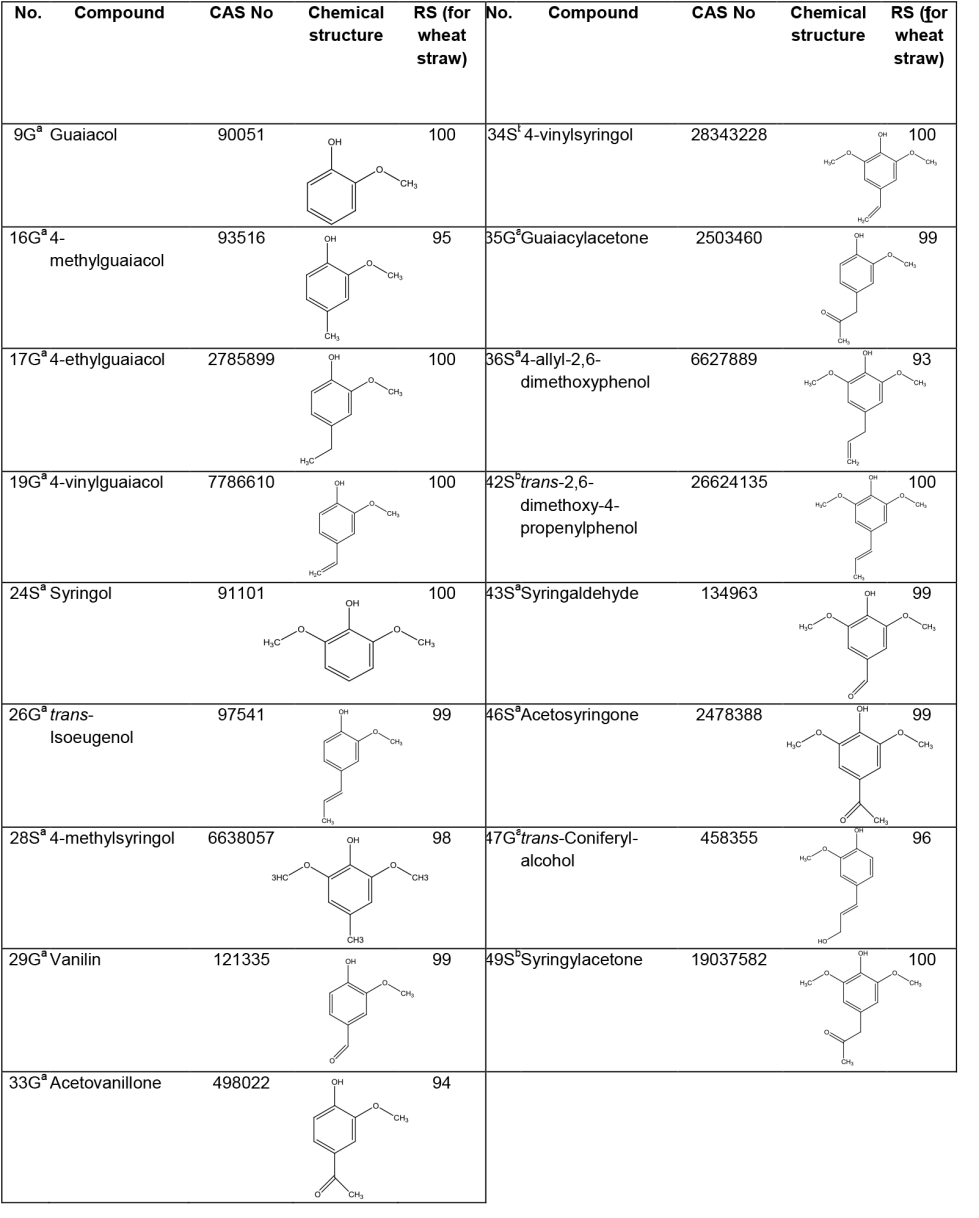
Schematic representation of sampling.

All samples were taken from the same original BM (same timeline). The first tunnel (35 x 4 x 4 meter) was filled with 200 tons of BM. Of this BM 100 kg was kept apart and divided into 3 batches (A, B, C) of about 33 kg. Each batch was handled separately and placed in onion mesh bags in the same tunnel (Fig 1). Per batch two bags (biological duplicates) were prepared and weighed (min 15 kg), labelled (e.g. A–1 and A–2) and placed over the length of the tunnel, about 30 cm below the surface of BM compost. After phase PI, both bags from the corresponding batch were weighed and afterwards thoroughly mixed, and one sample (min 1 kg) was taken from each batch (A, B and C). After sampling, the material was again mixed and divided over two bags and placed in the tunnel for phase PII (35 x 4 x 3.62 meter). Total compost in PII tunnel was 200 tons. The same procedure was followed for phase PIII (tunnel 35 x 4 x 3.62 meter, total compost 144 tons). So, for each sampling step (end of each phase) three samples (biological triplicates) were obtained (min 1 kg). Throughout the complete composting process, the batches (A, B and C) were weighed at the end of each phase and all the changes (addition of water, spawn) were noted. Weight of each sample (batch) was determined after sampling and after collecting, samples were immediately frozen at -18°C. First, the dry matter content was determined for each sample (100 g, 105°C overnight). From the fresh weight of each batch the dry matter yield was determined for PI, PII and PIII–16. The dry matter yield was 91.9% of PI, of PII 77.1% and of PIII–16 69.4% (average of three batches, STDEV 1.2, 1.9 and 1.9, respectively). Dried samples were milled (<1 mm) using an MM 2000 mill (Retsch, Haan, Germany) prior to further analysis. Samples were analyzed for their protein, carbohydrate, ash and lignin contents for each sample (batch). Contents of all analyzed components was summed up and compared to the dry matter content of corresponding sample and the recovery was found to be >95%. In addition, the carbohydrate and lignin composition was analyzed.

### Preparation of water un-extractable solids (WUS)

For batch A, freeze dried, milled samples (5 g) of BM, PI, PII and PIII–16 was suspended in water (175 mL) and boiled at 100°C for 5 min. Next, the suspension was stirred for 16 h at 21°C. The supernatant was removed after centrifugation (10 000 x g, 30 min, 20°C) and the residue was washed twice with water (60 mL and 75 mL). The final residues were freeze dried and collected as water un-extractable solids (e.g. PI-WUS). Samples were analyzed for yield, protein and dry matter contents.

### Analytical techniques and methods

#### Carbohydrate content and composition

The neutral carbohydrate and uronic acid content and composition was determined in duplicate, as described by Jurak et al. [2].

#### Nitrogen and protein content

Samples (7–10 mg) were analyzed for nitrogen content in duplicate using the combustion (DUMAS) method on a Flash EA 1112 Nitrogen Analyzer (Thermo Scientific, Sunnyvale, CA, USA). Methionine (Acros Organics, Geel, Belgium) was used as a standard. Nitrogen content in the water soluble extract was calculated by difference. Nitrogen to protein conversion factor of 6.25 was used [12]. For PIII–16, due to the presence of *A*. *bisporus* mycelium in compost, protein was not specified (n.s. Table 1).

**Table 1:** Mass balance of dry matter and organic matter and structural components (carbohydrates, nitrogen, lignin and ash) in the compost during composting and mycelium growth. BM: basic mixture; PI: compost after Phase I; PII: compost after Phase II; PIII–16: compost after 16 days of mycelium growth for batch A.

			BM	PI	PII	PIII–16
kg	Total dry matter		1000	914	787	708
	Carbohydrates	Total saccharides	439±9	412±7	197±16	186±1
		Mannitol	0	0	0	3
	Nitrogen	Water insoluble nitrogen	8±0.3	8±0.1	11±1.4	15±0.5
		Insoluble protein^c^	48±2	47±0.6	66±8.6	n.s.
		Water sobluble nitrogen^d^	6±0.3	6±0.0	5±1.4	1±0.1
		Total nitrogen	14	14	16	16
	Lignin	Klason lignin^e^	212±44	209±63	188±10	153±6
		Acid soluble lignin	49±23	44±13	64±19	61±23
		Total lignin	261	253	252	214
	Ash		199±1	215±2	236±1	240±5
	Organic matter (OM)^a^		801	699	551	468
% w/w	Loss OM relative to BM		-	13	31	42
	Loss dry matter relative to BM		-	9	21	29
	Recovery^b^		95	100	95	100

^a^ Organic matter = Total dry matter–ash.

^b^ Recovery = Calculated as sum(carbohydrates+protein+total lignin +ash)/total dry matter*100.

^c^ Nitrogen to protein conversion factor 6.25.

^d^ Calculated as difference = total nitrogen-water insoluble nitrogen.

^e^ Corrected for ash.

n.s. not specified. +/- STDEV between analytical duplicates.

#### Ash content

Freeze dried samples (1 g) or lignin residues (200–400 mg; see 2.4.4.) were dried in the oven overnight (105°C) and weighed, then put at 575°C for 5 h. Next, samples were weighed and difference between the mass at 105°C and 575°C was taken as ash content. Additionally, samples were burned at 575°C for 16 h more and afterwards weighed. No difference in mass was observed between residue after 5 h and 21 h.

#### Klason lignin residue and acid soluble lignin (ASL)

To each sample of 1 g (dry matter) 10 mL of 72% w/w H_2_SO_4_ was added and samples were hydrolyzed for 1h at 30°C. Next, 100 mL of distilled water was added to each sample and samples were put in a boiling water bath for 3h and shaken every half hour. Next, the suspensions were filtered over G4 glass filters. The filtrate was measured for acid soluble lignin (ASL) spectrophotometrically at 205 nm. ASL was calculated according to the formula: ASL = (A * B * C)/(D * E), with A = absorption relative to 1M H_2_SO_4_, B = dilution factor, C = filtrate volume, D = extinction coefficient for lignin (110 g L^−1^ cm^−1^), and E = weight of substrate (g). The residual part was washed until it was free of acid (determined by using pH paper) and dried overnight at 105°C. The final residues were corrected for ash and considered as a measure for the acid insoluble lignin (Klason) content after ash-correction. To this end, acid insoluble lignin was burned for ash. Total lignin was defined as a sum of Klason lignin residue, corrected for ash, and acid soluble lignin. For wheat straw, Klason lignin content corrected for ash was 27% (w/w) and acid soluble lignin content was 1.9% (w/w) based on dry matter.

#### Lignin analysis by analytical pyrolysis-GC-MS (Py-GC/MS)

**Fig 2:**
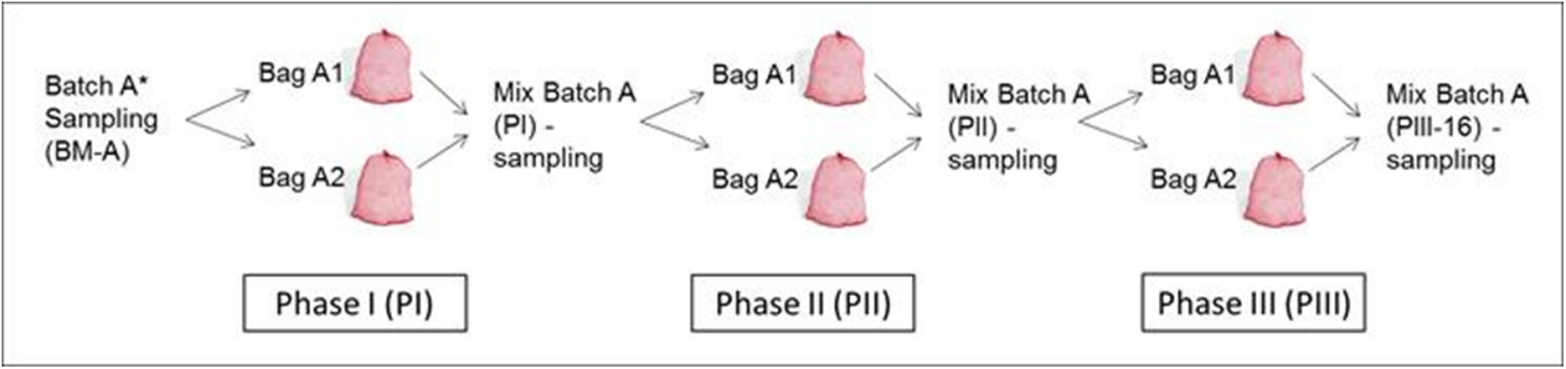
Identities of lignin-derived phenolic S (syringyl-like) and G (guaiacyl-like) compounds identified with Py-GC/MS and relative molar area higher than 1% in wheat straw (out of total S+G molar area). ^a^Interpretation based on pure compounds. ^b^Interpretation based on Ralph and Hatfield (1991), reverse search of compound in compost or WUS versus compound in wheat straw: 34S>99%, 42S>97%, 49S>99%. RS = reverse search

Pyrolysis was performed with a 2020 microfurnace pyrolyzer (Frontier Laboratories, New Ulm, MN, USA) equipped with an AS-1020E Autoshot. Components were identified by GC-MS using a Trace GC equipped with a DB–1701 fused-silica capillary column (30 m x 0.25 mm i.d. 0.25 μm film thickness) coupled to a DSQ-II (EI at 70 eV) (both Thermo Scientific, Waltham, MA, USA). The pyrolysis was performed at 500°C for 1 min. Helium was the carrier gas (1 mL min^−1^). Samples (60–70 μg) were pyrolyzed and each measurement was performed at least in triplicate. Initial oven temperature was 70°C (2 min hold) and it increased to 230°C with a rate of 5°C min^−1^, to 240°C by 2.5°C min^−1^ and finally to 270°C min^−1^ by 2.5°C min^−1^. Pure compounds were used as standards (Sigma Aldrich, St. Louis, MO, USA; Brunshwig Chemie B.V., Amsterdam, The Netherlands and Fisher Scientific, Landsmeer, The Netherlands) and peak molar area was calculated as defined by del Rio [13]. For wheat straw a cut-off of 1% molar area for single S (syringyl-like lignin structures) and G (guaiacyl-like lignin structures) compounds was applied and only the fate of remaining compounds (>1% molar area) was analyzed for compost samples. Compounds with a molar area >1% in wheat straw are specified in Fig 2. For WUS, the fate of the same S and G compounds as in original compost was compared. Remaining S and G compounds were annotated as Rest S* and Rest G*. The same cut-off level was applied for phenolic furanose/pyranose (F/P) and unknown compounds based on total area of these compounds. F/ P compounds with a molar area >1% are annotated in S1 Table. The remaining compounds are specified in S2 Table. Amdis software (version 2.71, NIST, USA) was used for identification and deconvolution of peaks. For deconvolution the following parameters were set: adjacent peak subtraction = one, resolution = medium, sensitivity = high and shape requirements = low. For identification a target compound library (based on referents standards) was built. Referents standards were measured in order to obtain retention time (RT) information and mass spectra (Fig 2, S1 Table and S2 Table). Compounds identified based on referents standards were, first, selected based on RT (± 1.0 min; or ± 0.1 min for isomers). If RT was within the selected window an annotation was given if reversed search (RS) value was higher than 80%. Finally, for all WS compounds, also the ones identified based on Ralph and Hatfield [14], spectra were checked manually. Total annotated area of S- and G- lignin units in wheat straw was ±80%.

#### Estimation of lignin quantities with analytical Py-GC/MS

To estimate absolute amounts of lignin in the samples, the areas of Py-GC/MS pyrograms were assumed to indicate amounts of lignin units present. As a base, the total lignin content (sum of Klason lignin (26.5% w/w) and ASL (1.9% w/w)) of wheat straw was correlated with the area under the Py-GC/MS pyrograms of wheat straw. Molar areas of S- and G-units annotated in Fig 2 and S2 Table were summed up as total molar area. For wheat straw and compost samples about 85% of dry matter was pyrolyzed in the Py-GC/MS, based on gravimetric analysis prior and after the pyrolysis. As the same amount of sample was weighed and pyrolyzed for wheat straw and compost samples it was assumed that correlation between wheat straw and compost lignin could be made. Also, in compost samples, lignin originates only from wheat straw. The correlation between total molar area of S and G with the w/w % of total lignin in wheat straw, was used to calculate the w/w % of lignin in compost samples based on the molar Py-GC/MS areas of the compost samples analysed. Lastly, obtained values for w/w % of lignin based on dry matter in compost samples was used to calculate the mass balance of lignin in PI, PII and PIII–16.

## Results and Discussion

### Dry matter, organic matter, carbohydrate and protein mass balance during composting and mycelium growth phases

The contents based on dry matter of carbohydrates, ash, Klason lignin residue (- ash) and nitrogen were analyzed for all three batches (A, B and C) are presented on a dry matter basis in Table 2. Previously, the composition of compost was reported for compost samples from BM, PI, PII and PIII–16 [2]. In that research, samples were obtained by mixing multiple compost samples from different tunnels with the aim to study the remaining carbohydrate structures, and a mass balance could not be performed. However, in order to fully understand the changes in the compost, a mass balance for dry matter as well as for proteins, cellulose, xylan, lignin and ash and, therefore, samples from the same timeline is needed. From Table 2, batch C was found to be an outlier with respect to carbohydrate content and dry matter content of Phase I (PI). Namely, in 1000 kg of basic mixture (BM), based on the carbohydrate content, 424 kg of carbohydrates were present compared to 440 kg in PI, for batch C. While 439 kg in BM and 412 kg in PI and 449 kg in BM and 420 kg in PI of carbohydrates, were calculated for batch A and B, respectively. So, only for batch C this would, impossibly, indicate a gain in carbohydrates in PI. Its carbohydrate content was analyzed at least 3 times indicating that this outlier was not due to an analytical error. Considering the correct values of PII and PIII–16, the error appeared to have occurred in the sampling after PI. Hence, after carbohydrate and ash analysis, batch C was excluded from further analysis. Nevertheless, to our opinion the values obtained for batch A and B give representative data for the mass balance, also, because the numbers obtained are very close to the yearly average mass balance values of CNC-C4C (personal communication with CNC-C4C).

**Table 2:** Carbohydrate, ash, nitrogen, Klason lignin and dry matter content (based on dry matter) for compost after PI, PII and PIII–16. BM: basic mixture; PI: compost after Phase I; PII: compost after Phase II; PIII–16: compost after 16 days of mycelium growth; A, B, C different batches.

	Carbohydrate content (% w/w DM)^a^	Ash content (% w/w DM)	Total nitrogen content (% w/w DM)	Water insoluble nitrogen) content (% w/w DM)	Klason lignin (-ash) content (% w/w DM)	DM (% w/w)
BM	44^b^	21^c^	1.3^d^	0.8^d^	21^f^	100
PI-A	45	24	1.4	0.8	22^f^	91.4
PI-B	46	21	1.4	0.8	n.a.	91.1
PI-C	47	22	n.a.	n.a.	n.a.	93.2
average (STDEV)	46 (1.0)	22 (1.5)	1.4 (0.03)^e^	0.8 (0.04)^e^	n.a.	91.9 (1.2)
PII-A	25	30	2.0	1.4	23^f^	78.7
PII-B	26	29	2.1	1.7	n.a.	74.9
PII-C	27	28	n.a.	n.a.	n.a.	77.7
average (STDEV)	26 (1.1)	29 (0.9)	2.1 (0.03)^e^	1.6 (0.2)^e^	n.a.	77.1 (1.9)
PIII-16-A	26	34	2.3	2.1	21^f^	70.8
PIII-16-B	27	30	2.2	2.1	n.a.	67.2
PIII-16-C	23	30	n.a.	n.a.	n.a.	70.2
average (STDEV)	26 (1.9)	3 (2.3)	2.2 (0.07)^e^	2.1 (0.5)^e^	n.a.	69.4 (1.9)

^a^Each sample was analyzed in duplicate (STDEV <1).

^b^Average of dupplicates of batches A, B and C (STDEV 1.3).

^c^Average of duplicates of batches A, B, C (STDEV 1).

^d^Average of dupplicates of batches A and B (STDEV 0.4).

^e^Average of duplicates of batches A and B.

^f^Average of duplicates for batch A (STDEV BM 0.4, PI 7, PII 1.3, PIII–16 1).

n.a. not analyzed; DM dry matter.

The carbohydrate contents (based on dry matter) of batch A and B was found to be, on average, 44% w/w for BM, 46% w/w for PI, 26% w/w for Phase II (PII) and 26% for PIII–16. Ash content was found to be 21%, 22%, 29% and 30% for BM, PI, PII and PIII–16, respectively (w/w based on dry matter). Total nitrogen content was 1.3%, 1.4%, 2.1% and 2.2% for BM, PI, PII and PIII–16, respectively and water insoluble nitrogen content was found to be 0.8%, 0.8%, 1.6% and 2.1% for BM, PI, PII and PIII–16, respectively (w/w based on dry matter). Lastly, Klason lignin contents, corrected for ash, were 21%, 22%, 23% and 21% for BM, PI, PII and PIII–16, respectively (w/w based on dry matter).

Next, for batch A the mass balance concerning ash, protein, carbohydrates and lignin during composting and mycelium growth is presented (Table 1) based on a starting amount of 1000 kg dry matter BM. The totals of all analyzed components covered 95% w/w or more of the total amount of dry matter, indicating the completeness of the analyses performed. Compared to BM, a decrease of 8% w/w of dry matter was analyzed for PI, 23% w/w for PII and 31% w/w for PIII–16.

Overall, some variations in the absolute amounts of ash was observed (Table 1). Previously, variations in the amount of inorganic materials during composting have been reported [1]. Ash present mainly originated from sand and stones found in the commercial compost solids. Possibly, these are introduced together with recycled process-water, and therefore present in various amounts in the different samples (personal communication CNC-C4C). Such ash-recycles may also contribute to the higher decrease in organic matter (OM) compared with DM (Table 1).

**Fig 3:**
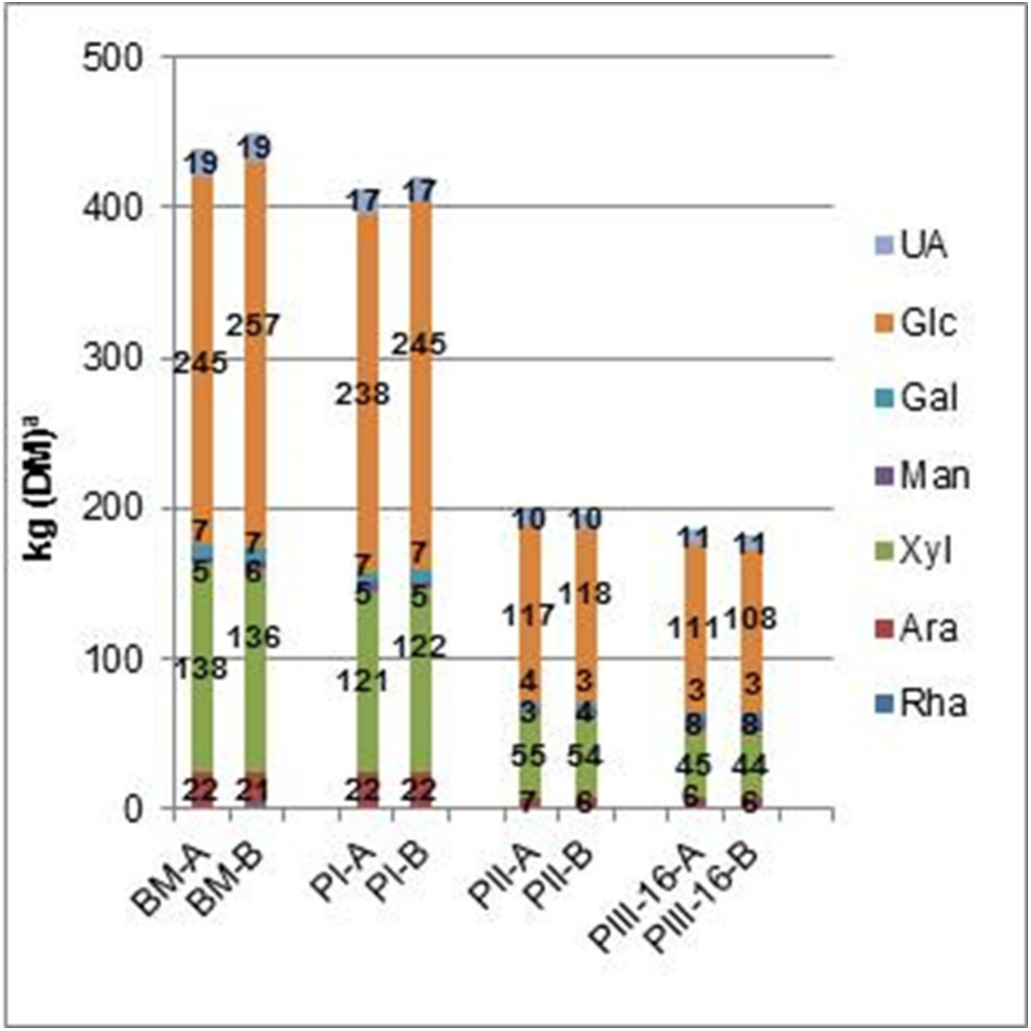
Mass balance of constituent monosaccharides during composting and mycelium growth for batch A and B (BM, PI, PII, PIII–16). BM = basic mixture, PI = compost after Phase I, PII = compost after Phase II, PIII–16 = compost after 16 days of mycelium growth; Rha = ramnosyl, Ara = arabinosyl, Xyl = xylosyl, Man = mannosyl, Gal = galactosyl, Glc = glucosyl, UA = uronyl. ^a^Calculation was performed based on 1000 kg of DM using values presented in Tables 1 and 4.

Total nitrogen remained rather similar in the compost during composting and mycelium growth. Given the low nitrogen values (% w/w, Table 2), comparison of absolute nitrogen amounts should be performed with caution. Given this, a tendency in increase of water insoluble nitrogen might be observed in PII compost compared to PI compost. During PI rise in temperature to 80°C [2] and formation of ammonia was observed (personal communication CNC-C4C) indicating microbial growth of meso- and thermophilic microbiota [6], however, no big differences in carbohydrate and lignin content were observed (Table 2). In contrast to this, a tendency in increase in the amount of total nitrogen and protein in PII (mass balance, Table 1) could be interpreted. During PII a decrease in the organic matter (18% w/w) and carbohydrates (±50% w/w for both xylan and cellulose present (Fig 3)) was observed. During this phase up to 40% of ammonia is reported to be removed by microbiota, actinomycetes and fungi, present [6] with temperatures around 50°C [2] and higher humidity compared to PI (Table 1). With caution it could be proposed that one of the possible explanations for this observation is the growth of nitrogen-fixating and other viable microbiota introduced into compost at the beginning of PII [15, 16].

In PIII–16 (Table 1) temperatures are maintained around 25°C [2] and humidity is higher (±8%) compared to PII. The amount of protein in PIII–16 increased further, mainly seen in the increase in water insoluble nitrogen, and the amount of carbohydrates decreased slightly both in xylan and cellulose (Fig 3), most likely as a result of the observed mycelium growth in this phase [17, 18]. It should be noted that due to the formation of mycelium dry matter, partly built from glucan, the decrease in compost-glucan (cellulose; Fig 3) derived from the starting material is underestimated. Namely, in our analysis, total glucan was analyzed, regardless whether it originated from plant or microbial origin. Finally, mannitol was analyzed to be present in PIII–16 compost, which is a known soluble carbohydrate in the mycelium of *A*. *bisporus* [19].

Overall, the molar composition of the compost carbohydrates in BM, PI, PII and PIII–16 (Table 3) remained rather similar and is in line with previously reported data [2]. However, a decrease in xylosyl residues could be observed in PIII–16 compared to PII. In all phases, the main carbohydrate constituents were xylosyl (28–35 mol%) and glucosyl (52–56 mol%) residues, which is in agreement with previously published data [2]. Recently, it was shown that during PIII compost xylan is partly degraded, thereby, making it more water soluble [2] which is expected to provide more easily accessible carbohydrates during fruiting of *A*. *bisporus*. In the present study, no division between water soluble and water insoluble glucans and xylan was performed.

**Table 3:** Carbohydrate composition (mol%) and degree of substitution of xylan in different compost phases, based on dry matter. BM: basic mixture; PI: compost after Phase I; PII: compost after Phase II; PIII–16: compost after 16 days of mycelium growth.

	Mol%^a^								
	Rha^b^	Ara^b^	Xyl^b^	Man^b^ ^c^	Gal^b^	Glc^b^	UA^b^	Ara/Xyl^d^	GlcA/Xyl^d^
BM-A	1	6	36	1	2	52	4	16	10
BM-B	1	5	35	1	1	53	4	15	10
BM-C	0	6	35	1	2	52	4	16	11
Average^e^	1	6	35	1	2	52	4	16	10
STDEV^e^	0.0	0.2	0.6	0.1	0.0	0.8	0.1	0.4	0.4
PI-A	1	6	33	1	2	54	4	18	11
PI-B	1	6	33	1	2	54	3	18	10
PI-C	1	6	33	1	1	55	3	17	10
Average^e^	1	6	33	1	2	54	3	17	10
STDEV^e^	0.0	0.2	0.1	0.1	0.1	0.4	0.1	0.5	0.4
PII-A	1	4	32	2	2	56	5	12	14
PII-B	1	4	32	2	2	56	4	12	14
PII-C	1	4	31	2	2	56	4	12	14
Average^e^	1	4	32	2	2	56	4	12	14
STDEV^e^	0.0	0.1	0.4	0.1	0.0	0.5	0.1	0.1	0.0
PIII-16-A	1	3	28	4	2	57	5	12	18
PIII-16-B	1	4	28	4	2	56	5	13	18
PIII-16-C	1	4	28	4	2	56	5	13	19
Average^e^	1	4	28	4	2	56	5	13	18
STDEV^e^	0.2	0.1	0.1	0.1	0.1	0.3	0.2	0.3	0.5

^a^Molar compostition. Rha = ramnosyl, Ara = arabinosyl, Xyl = xylosyl, Man = mannosyl, Gal = galactosyl, Glc = glucosyl, UA = uronyl.

^b^Carbohydrate analysis in duplicate, average of duplicates presented, STDEV within samples in range from 0.1 to 1.

^c^Not corrected for mannitol.

^d^Ratio mol/100mol.

^e^Average and standard deviation (STDEV) batch A, B and C.

### Lignin mass balance during composting and mycelium growth phases

First, the Klason lignin analysis was applied to a lab-cultivated *A*. *bisporus* mycelium sample, which allowed us to observe the fate of mycelium in this analysis. It was shown that more than 50% of the mycelium dry matter, was collected as ‘Klason lignin’. This indicated that the Klason lignin analysis in samples containing substantial amounts of mycelium, like in PIII–16, would give an overestimation of the lignin present as mushroom mycelium would be analyzed as “lignin” by this method. Also, denatured proteins are known to remain in the Klason lignin residues [1]. Nevertheless, Klason lignin residues were analyzed for batch A (Table 1), allowing comparison with the scarce previously reported data on compost composition [1]. For PI and PII values obtained for Klason lignin corrected for ash were in line with values reported by Iiyama et al. [1].

Lignin structure and content was also analyzed as single monolignol units by analytical Py-GC/MS. Based on the correlation between the wheat straw Klason lignin content, and the area of annotated S- and G-units in the wheat straw pyrogram obtained, from the pyrogram-areas of the compost samples (in triplicate) the lignin yield in these samples was calculated, based on 1000 kg BM dry matter (Table 4). In general, the amount of pyrogram based lignin remained rather similar during composting (PI and PII). In contrast, a decrease of 45% w/w in the amount of lignin, based on pyrolysis, was observed after 16 days of mycelium growth. The overall difference in kg between BM and PIII–16 is more pronounced by the GC/MS analysis than by the classical Klason lignin analysis. This also accounts for the decrease in dry matter during the PIII phase. In our opinion, the Py-GC/MS data are more representative for the lignin amounts present than the Klason lignin residue analysis, because with the former technique only lignin derived pyrolysis units were taken into account in the quantification in mycelium grown compost samples. The GC/MS analysis leads to the total lignin yield based on constituent units present after pyrolysis. Hence, this technique also provided valuable data on compositional changes during the different phases.

**Table 4:** Relative pyrogram area (%) and Py-GC/MS pyrogram based lignin for wheat straw and different compost phases for batch A. BM: basic mixture; PI: compost after Phase I; PII: compost after Phase II; PIII–16: compost after 16 days of mycelium growth.

Sample	Relative pyrogram area (%)^a^	Lignin (pyrogram based) yield (kg)^b^
Wheat straw	100	284±4^c^
BM-A	86±7	245±28
PI-A	70±7	172±21
PII-A	90±8	208±36
PIII-16-A	45±3	91±4

^a^Total of areas of all S- and G-units annotated in Table 1 and S1 Table.

^b^Based on 1000 kg dry matter BM; calculated based on wheat straw total lignin and pyrogram analysis.

^c^Lignin yield for wheat straw based on Klason lignin+acid soluble lignin. STDEV between duplicates.

±STDEV between triplicates.

### Structural changes of lignin during composting and mycelium growth phase

**Fig 4:**
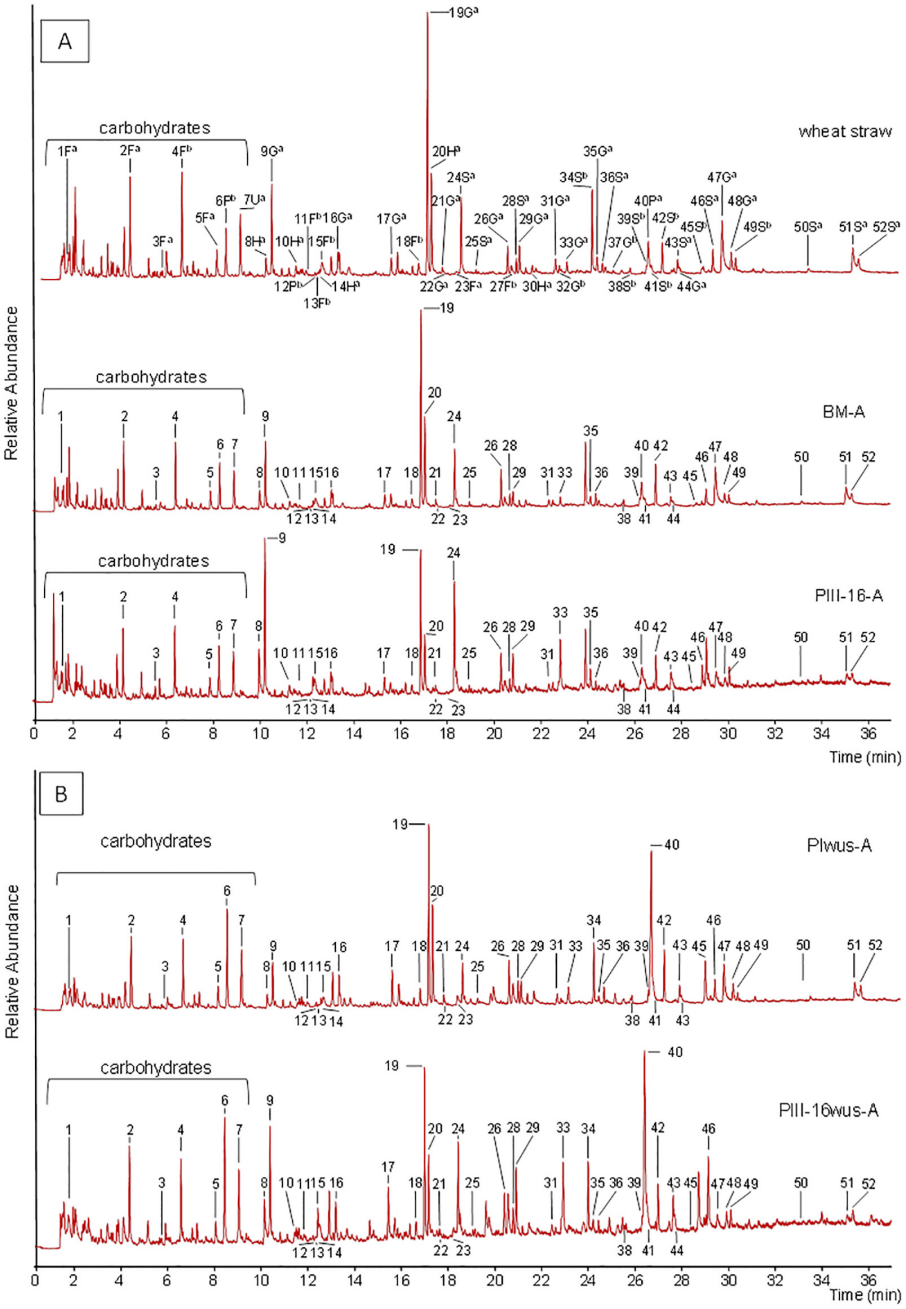
Py-GC/MS pyrograms of wheat straw, basic compost mix (BM) and compost after 16 days of mycelium growth (PIII–16) (A) and water un-extractable solids (WUS) of Phase I and PIII–16 (B) for batch A. The identities and structures of main syringyl and guaiacyl (and p-hydroxyphenyl) compounds are listed in Fig 2, S1 Table and S2 Table. PI: compost after Phase I: PII: compost after Phase II.

The Py-GC/MS lignin-fingerprints of the BM, PI, PII and PIII–16 composts were annotated based on the fully annotated pyrogram of untreated wheat straw (Fig 4A). The pattern and main annotated peaks of lignin compounds for wheat straw were in line with previously reported data [4]. Due to better baseline separation and additional spectra measured from standard lignin compounds, some peaks (e.g. trans-isoeugenol, 4-methylsyringol, vanilin) were differently annotated than previously reported (Fig 2, S1 Table and S2 Table) [4].

For BM the pattern and the ratios between peaks is quite similar to those of wheat straw, which was expected as lignin in BM-compost originates from wheat straw. Also, the pyrograms of PI and PII composts were majorly similar as the ones of BM and wheat straw. In Phase III, however, the ratios (in molar area) between some monolignol-units, mainly vinyl-guaiacol, guaiacol, vinyl-syringol and syringol, were very different between the BM and PIII–16 pyrograms (Fig 4).

In order to understand the differences observed during mycelium growth, first, the various monolignol-units present in the pyrogram of wheat straw are discussed. As previously stated, wheat straw lignin is mainly composed of S- (syringyl-like) and G- (guaiacyl-like) lignin units, and to a minor extent of H (p-hydroxyphenyl) units. Therefore, we focused on S and G lignin units.

**Fig 5:**
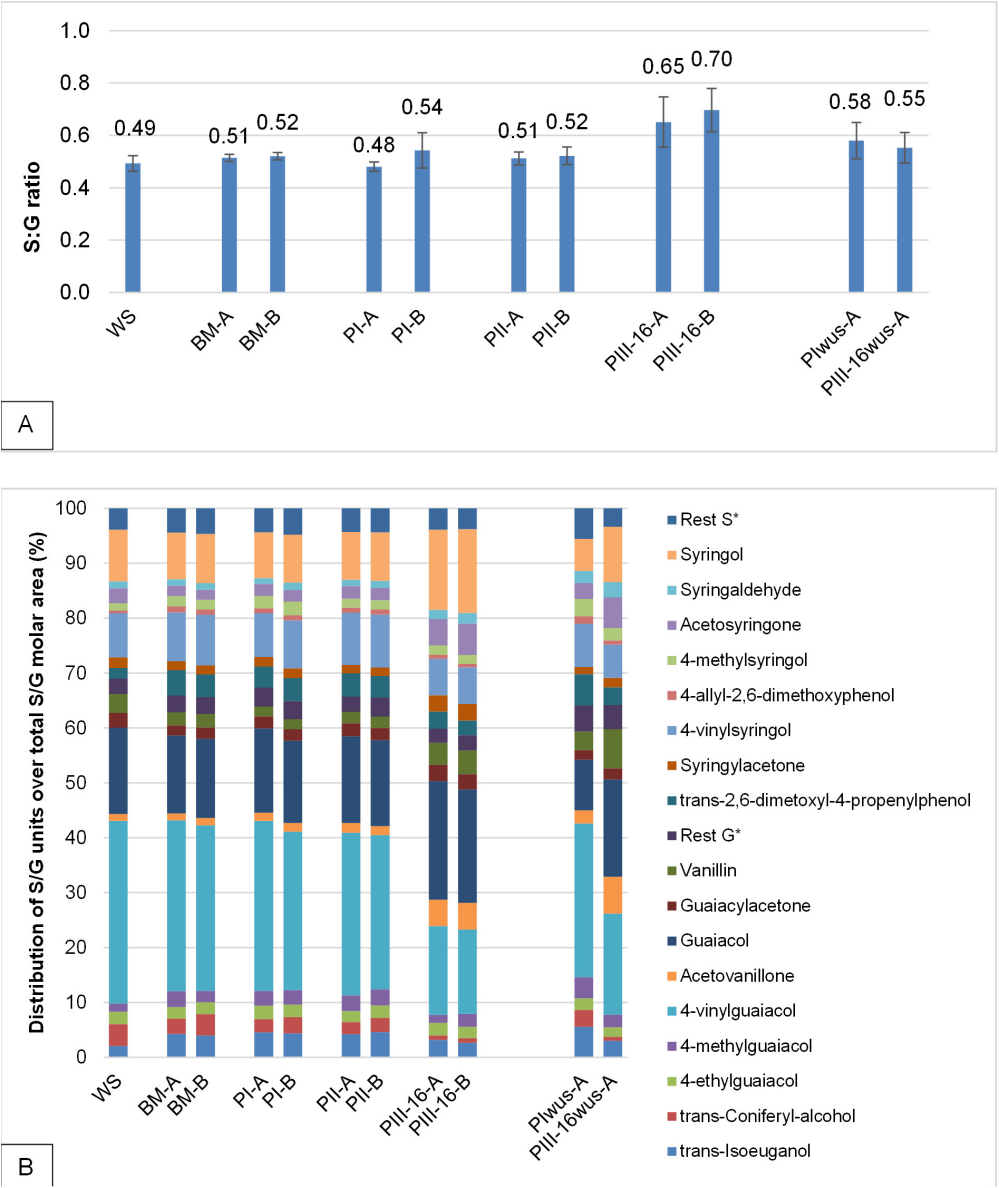
S:G ratio (A) and distribution of syringyl (S) and guaiacyl (G) units (B), based on molar area, of wheat straw (WS) and total compost samples during composting BM, PI, PII and mycelium growth PIII–16 and in water insoluble compost PIwus, PIII-16wus. Rest S* and Rest G* S2 Table, BM: basic mixture; PI: compost after Phase I: PII: compost after Phase II; PIII–16: compost after 16 days of mycelium growth, A and B are biological duplicates and each sample measurement was performed in quadruplicates.

The S:G ratios of wheat straw and different compost samples was calculated and is shown in Fig 5A. The S:G ratio in wheat straw was 0.49 (Fig 5A), which is in line with the value reported by del Rio [4], where vinyl-syringol and vinyl-guaiacol were excluded from the S:G ratio. The S:G ratio in PI and PII remained 0.51. After 16 days of mycelium growth (PIII–16), the S:G ratio changed to 0.68 (Fig 5A), indicating a modification in lignin by *A*. *bisporus* mycelium. The pyrograms obtained for compost samples as well as S:G values are found to be in line with values reported by Chen et al. [20] however, annotated peaks were not specified and the samples used were collected from different stages in the process. Therefore, these results are difficult to compare.

Changes in distribution of S and G lignin units were determined for S and G structures with molar area larger than 1% of total S+G molar area (Fig 2). Remaining annotated compounds are presented in S1 Table and S2 Table (see materials and methods), but not taken into account further.

Compared to PII compost, a lower proportion of substituted vinyl-syringyol and vinyl-guaiacol lignin compounds in PIII–16 compost was present in favor of the less substituted guaiacol and syringol (Figs 2 and 4). This may point at cleavage of substituents on the phenolic skeleton during PIII (Fig 5B). The observed modification of substituents is mainly observed in “vinyl- groups” leading to a relative decrease in vinyl-guaiacol and vinyl-syringol during PIII. Lignin structures analyzed by NMR in wheat straw [4] indicate that such vinyl-decorations are mainly responsible for inter-lignin linkages. Therefore, our findings suggest that *A*. *bisporus* is capable of cleaving larger lignin structures into smaller ones, and further remove the decorations leaving mainly the basic S and G phenolic skeletons of the lignin structures.

In previous research [4], vinyl-guaiacol and vinyl-syringol were excluded from the S:G ratio as during pyrolysis p-hydroxycinnamates are known to result in the same compounds as those derived from lignin. If these compounds were only part of xylan, it could lead to overestimation of lignin. However, in BM, due to high pH, no free and ester bound FA (ferulic acid) and very low amounts of *p*CA (p- coumaric acid) (<0.1% w/w based on dry matter) were found [2]. This indicated that in BM compost less than 0.3% (w/w based on dry matter) of ester bound FA and *p*CA were present. Also, the amount of vinyl-guaiacol that could be formed from FA and *p*CA after pyrolysis was less than 4% of the total of vinyl-guaiacol analyzed in the wheat straw pyrogram. The remaining ether-bound FA and *p*CA are expected to account for less than 0.5% w/w based on dry matter [21].

Composition and S:G ratio of water insoluble lignin in compost samples is presented in Fig 5 and corresponding pyrograms are presented in Fig 4. As no major compositional changes in the relative distribution of S and G lignin compounds were observed between BM and PII (Fig 5B), only for PI (PIwus) and PIII–16 (PIII-16wus) water insoluble lignin was analyzed in detail in particular for batch A. For PI the distribution of S and G compounds of PIwus-A (Fig 5A) was found to be rather similar as that of the total sample (PI-A). On the contrary, in PIII-16wus a relatively lower S:G ratio was found compared to the total sample of PIII–16 indicating that part of lignin in PIII–16 compost became more water soluble (Fig 5B).

Lignin modification in the compost by *A*. *bisporus* mycelium was previously indicated based on the degradation on ^14^C-labelled lignin [22] and *A*. *bisporus* was shown to produce the with lignin degradation correlated activities of manganese peroxidase and laccase in liquid cultures [23]. Recently, annotation of the *A*. *bisporus* genome indeed showed that *A*. *bisporus* contains genes encoding manganese peroxidase (MnP) and laccases [24, 25]. Regulation and expression of laccases and MnP was investigated and two genes encoding laccases and the predicted MnP gene were found to be highly expressed during mycelium growth in the compost and lower expression in the later stages of mushroom growth. In addition, the secretion of corresponding proteins indicated that laccases are secreted to a higher extent compared to MnP [24, 26]. These findings support the data presented in our research showing that *A*. *bisporus*, during the vegetative growth, was able to modify lignin structures. It is proposed that observed lignin degradation and modification increase the bioavailability of the carbohydrates in the wheat based compost.

To our knowledge, this is the first time that degradation and metabolization of lignin by *A*. *mycelium* was shown directly on the lignin structure in mycelium grown wheat straw based compost. Overall, our research provides more insights in how *A*. *bisporus* mycelium degraded lignocellulosic biomass for mushroom growth, and in general, give new insights in lignocellulosic plant biomass degradation.

## Conclusion

During PI of composting, no changes in carbohydrates and lignin were observed in the compost. In PII, 50% of carbohydrates, both cellulose and xylan were metabolized, while lignin structure was not. During 16 days of mycelium growth (PIII–16) 45% of lignin was metabolized and the remaining lignin was modified resulting in an increased S:G ratio (0.51 to 0.68). Furthermore, from both S and G phenylpropanoid units the decorations, mainly vinyl-groups, were removed from the phenolic skeleton.

## Supporting Information

S1 Table Identities of lignin-derived phenolic F/P and unknown compounds identified with Py-GC/MS with relative molar area higher than 1% in wheat straw (out of total F/P and unknown compounds molar area) (DOCX)

S2 Table Identities of lignin-derived phenolic S, G, F/P and unknown compounds below 1% of relative molar area in wheat straw (for S and G out of total S+G molar area, and for F/P and unknown out of total F/P + unknown molar area) identified with Py-GC/MS. (DOCX)
