# Supplementary material for: Fate of Carbohydrates and Lignin during Composting and Mycelium Growth of Agaricus bisporus on Wheat Straw Based Compost
Source: PLoS One. 2015 Oct 5;10(10):e0138909. doi: 10.1371/journal.pone.0138909 (PMC4593547; doi:10.1371/journal.pone.0138909)
Supplement: S1 Table — (DOCX) [file pone.0138909.s001.docx]

S1 Table. Identities of lignin-derived phenolic F/P and unknown compounds identified with Py-GC/MS with relative molar area higher than 1% in wheat straw (out of total F/P and unknown compounds molar area).

| No. | Compound | CAS number | Chemical structure | RS  (for wheat straw) | No. | Compound | CAS number | | Chemical structure | RS  (for wheat straw) |
| --- | --- | --- | --- | --- | --- | --- | --- | --- | --- | --- |
| 1F^a^ | 2-methylfuran | 534225 |  | 99 | 10H^a^ | O-cresol | 95487 | |  | 99 |
| 2F^a^ | Furfural | 98011 |  | 100 | 12P^b^ | 2,4-dihydropyran-3-one | - | |  | 100 |
| 3F^a^ | 2-acetylfuran | 1192627 |  | 100 | 14H^a^ | P-cresol | 106445 | |  | 97 |
| 4F^b^ | 2,3-dihydro-5-methylfuran-2-one | 591128 |  | 99 | 15F^b^ | 5-(hydroxymethyl) dihydro-2(3H)-furanone | 3278066 | |  | 100 |
| 5F^a^ | 2(5H)-furanone | 497234 |  | 100 | 18F^b,c^ | 1,4-anhydroxylofuranose | | - |  | 100 |
| 6P^b^ | 4-hydroxy-5,6-dihydro-(2H)-pyran-2-one | - |  | 100 | 20H^a^ | 4-vinylphenol | 2628173 | |  | 100 |
| 7U^a^ | 2-hydroxy-3-methyl-2cyclopenten-1-one | 765708 |  | 97 | 40P^a^ | 1,6-anhydro-β-D-glucopyranose | 498077 | |  | 100 |
| 8H^a^ | Phenol | 108952 |  | 99 |  |  |  |  |  |  |

^a^Interpretation based on pure compounds

^b^Interpretation based on Ralph and Hatfield (1991), reverse search of compound in compost or WUS versus compound in wheat straw: 6P>99%, 12P>87%, 15F>89%, 18F>99%.

^c^Compound 18F and 27F has similar spectra, assignment were based on relative abundance in the sample.

RS= reverse search
