# Supplementary material for: Fate of Carbohydrates and Lignin during Composting and Mycelium Growth of Agaricus bisporus on Wheat Straw Based Compost
Source: PLoS One. 2015 Oct 5;10(10):e0138909. doi: 10.1371/journal.pone.0138909 (PMC4593547; doi:10.1371/journal.pone.0138909)
Supplement: S2 Table — (DOCX) [file pone.0138909.s002.docx]

S2 Table. Identities of lignin-derived phenolic S, G, F/P and unknown compounds below 1% of relative molar area in wheat straw (for S and G out of total S+G molar area, and for F/P and unknown out of total F/P + unknown molar area) identified with Py-GC/MS.

| No. | Compound | | CAS No | Chemical structure | RS  (for wheat straw) | No. | Compound | CAS No | Chemical structure | RS  (for wheat straw) |
| --- | --- | --- | --- | --- | --- | --- | --- | --- | --- | --- |
| 11F^b^ | 5-ethyl-2-furfural | 23074104 | |  | 100 | 37G^b^ | Propiovanillone | 1835149 |  | 100 |
| 13F^b^ | Unknown, 2-acetoxy-5-ethylfuran? | 24241847 | |  | 97 | 38S^b^ | *cis*-2,6-dimethoxy-4-propenylphenol | 26624135 |  | 94 |
| 21G^a^ | Eugenol | 97530 | |  | 95 | 39S^b^  41S^b^ | 1-(3,5-dimethoxy-4-hydroxyphenyl) propyne | - |  | 93 |
| 22G^a^ | 4-propylguaiacol | 2785877 | |  | 92 | 44G^a^ | *cis*-Coniferyl-alcohol | 458355 |  | 99 |
| 23F^a^ | 5-hydroxy-  methylfurfural | 67470 | |  | 89 | 45S^b^ | Homosyringalde-hyde | - |  | 99 |
| 25S^a^ | *cis*-Isoeugenol | 97541 | |  | 98 | 48G^a^ | *trans*-Coniferaldehyde | 458366 |  | 99 |
| 27F^b,c^ | 1,4-anhydro-arabinofuranose | | - |  | 98 | 50S^a^ | *cis*-Sinapyl-alcohol | 537337 |  | 100 |
| 30H^a^ | Hydroquinone | 123319 | |  | 99 | 51S^a^ | *trans*-Sinapyl-alcohol | 537337 |  | 100 |
| 31G^a^ | 4-propylguaiacol | 2785877 | |  | 96 | 52S^a^ | *trans*-Sinapaldehyde | 4206580 |  | 95 |
| 32G^b^ | 4-ethyl-syringol | 14059928 | |  | 100 |  |  |  |  |  |

^a^Interpretation based on pure compounds

^b^Interpretation based on Ralph and Hatfield (1991), reverse search of compound in compost or WUS versus compound in wheat straw: 11F>92%, 13F>87%, 32G>97%, 37G>83%, 38S>88%, 39S/ 41S>82%, 45S>80%.

^c^Compound 18F and 27F has similar spectra, assignment were based on relative abundance in the sample.

RS= reverse search.
